# Adjusting to life after treatment: distress and quality of life following treatment for breast cancer

**DOI:** 10.1038/sj.bjc.6604091

**Published:** 2007-11-13

**Authors:** E S Costanzo, S K Lutgendorf, M L Mattes, S Trehan, C B Robinson, F Tewfik, S L Roman

**Affiliations:** 1 Department of Psychology, University of Wisconsin-Madison, Madison, WI, USA; 2 Departments of Psychology, Urology, and Obstetrics and Gynecology, University of Iowa, Iowa City, IA, USA; 3 Department of Psychology, University of Iowa, Iowa City, IA, USA; 4 Aultman Hospital, Canton, OH, USA; 5 Trinity Medical Center, Rock Island, IL, USA; 6 Iowa City Cancer Treatment Center, Iowa City, IA, USA; 7 Department of Internal Medicine, University of Iowa, Iowa City, IA, USA

**Keywords:** breast cancer, cancer survivorship, depression, anxiety, quality of life

## Abstract

Clinical and anecdotal findings suggest that the completion of cancer treatment may be marked by heightened distress and disrupted adjustment. The present study examined psychological adjustment during the 3 months following treatment among 89 women with stages 0–III breast cancer. Participants completed measures of depression, cancer-related anxiety, cancer concerns, and quality of life at three time points: during treatment, 3 weeks following the end of treatment, and 3 months post-treatment. Post-treatment scores were suggestive of good psychological adjustment among the majority of women. Moreover, distress did not increase following treatment; longitudinal analyses showed no significant changes in depression or recurrence worry, while intrusive thoughts decreased, and quality of life improved. Younger age predicted greater distress across measures. A history of depression or anxiety predicted greater depressive symptomatology, while more extensive treatment predicted greater cancer-related anxiety. Despite the lack of distress endorsed on general depression and anxiety indices, participants reported moderate distress associated with cancer-related concerns, including physical problems, fear of cancer recurrence, and resuming normal life. In sum, while breast cancer survivors demonstrate good adjustment on general distress indices following treatment, some women are at risk for sustained distress. Moreover, significant cancer-related concerns are prevalent and may be important intervention targets.

Breast cancer survivors appear to be remarkably well adjusted as compared to other women (Moyer and Salovey, 1996; Dorval *et al*, 1998; Ganz *et al*, 1998). However, there may be periods of disrupted adjustment during the survivorship experience. The completion of adjuvant treatment (chemotherapy and/or radiation therapy) may be one such period. Case studies and other anecdotal evidence in the literature suggest that the months immediately following the end of adjuvant treatment is a time of disruption, transition, and increased distress (Ward *et al*, 1992; Lethborg *et al*, 2000; McKinley, 2000; Schnipper, 2001).

There are several reasons why the post-treatment period may be a particularly distressing time for breast cancer survivors. First, women are still contending with the physical effects of treatment, including fatigue, hair loss, early menopausal symptoms, lymphoedema, and decreased libido. Many women become distressed because they had not anticipated ongoing treatment-related problems (Beisecker *et al*, 1997; Stanton *et al*, 2005). Second, cancer survivors no longer need to focus intensely on medical treatment, leaving room for a psychological struggle (Schnipper, 2001). Interviews with breast cancer survivors after the end of treatment revealed that women were just beginning to reflect on fears and existential issues at this time (Lethborg *et al*, 2000). Third, loss of support from family and friends who may not realise that cancer survivors continued to struggle with cancer-related physical and psychological issues is another potential source of distress (Stanton *et al*, 2005). The loss of regular contact with health-care providers may also result in feelings of decreased support (Lethborg *et al*, 2000).

A theme that resonates throughout the literature is the loss of a safety net: 29% of breast cancer patients who had received chemotherapy reported feeling as if a safety net had been lost once treatment ended (Ward *et al*, 1992). Regularly receiving treatment and attending medical appointments provides an active means for destroying existing cancer and preventing cancer growth and recurrence. Once treatment is over, this active coping strategy is no longer available. Fear of a cancer recurrence, the primary concern for 39% of women enrolled in a post-treatment intervention study (Stanton *et al*, 2005), coupled with the loss of one's primary means for managing cancer, may be a particularly distressing combination for cancer survivors (Lethborg *et al*, 2000).

Much of the evidence regarding disrupted adjustment during the months following treatment completion is anecdotal (Mullan, 1985; McKinley, 2000), and health-care providers have also described clinical observations of increased distress during this time (Rowland and Holland, 1990; Schnipper, 2001). Other studies have obtained information regarding distress via structured interviews of small patient samples. In these studies, 30–35% of breast cancer patients reported distress associated with the end of treatment (Ward *et al*, 1992; Beisecker *et al*, 1997). These results contrast with a single empirical study, which found that women completing radiation therapy for breast cancer experienced low levels of anxiety and depression during the 6 months following the end of treatment (Deshields *et al*, 2005).

While anecdotal and qualitative evidence is valuable in understanding women's experiences following treatment, the paucity of empirical studies of larger samples of breast cancer patients focused on post-treatment psychological adjustment limits the ability to generalise to a broader population of breast cancer survivors. Moreover, previous studies have not directly compared distress at the end of treatment to distress during treatment, and therefore, it is unknown whether the distress reported following treatment represents an increase or simply maintenance of previous distress. Finally, little is known regarding individual differences that predict which breast cancer survivors are most likely to become distressed following treatment.

The primary objective of the present study was to empirically examine the extent to which adjustment was disrupted during the months following the completion of treatment for breast cancer. Previous work is most suggestive of disrupted adjustment during the first 1–3 months following cancer treatment (Ward *et al*, 1992; Deshields *et al*, 2005) with cancer survivors showing nearly normal levels of adjustment 4–6 months after treatment ends (Andersen *et al*, 1989; Ward *et al*, 1992). Therefore, distress and quality of life were assessed towards the end of adjuvant chemotherapy and/or radiation treatment, 3 weeks after the end of treatment, and 3 months after the end of treatment. We hypothesised that anxiety and depressive symptoms, as well as cancer-related worry, would increase from mid-treatment to 3 weeks post-treatment, and decrease from 3 weeks post-treatment to 3 months post-treatment, while health-related quality of life would improve over time.

The later half of adjuvant treatment was selected as the baseline assessment time point, because it allowed for maximal adjustment to treatment and one's diagnosis. There is evidence that distress is quite high around the time of diagnosis, but there is a well-documented decline in distress over the course of adjuvant treatment (Ward *et al*, 1992; Osowiecki and Compas, 1999; Frost *et al*, 2000). We chose our baseline evaluation at a time in which distress was least likely to be elevated, which allowed for a comparison of distress levels after treatment with those during the routine of treatment.

A second objective of the study was to investigate sources of distress by assessing participants' concerns. It was predicted that concerns about cancer status, loss of a ‘safety net’, physical problems related to cancer and treatment, difficulty returning to ‘normal’, and loss of support from health-care providers and others would be prevalent. The third objective of the study was to identify demographic, psychiatric, and disease- and treatment-related predictors of post-treatment distress and distress trajectories.

## MATERIALS AND METHODS

### Participants

Participants were women treated with adjuvant chemotherapy, radiation therapy, or both for stages 0–III breast cancer. Women with recurrent or metastatic cancer were excluded. Eligible breast cancer patients (*N*=113) were approached to participate in the study at five treatment centres in the Midwest after approval from each institution's human subjects review board. Of those approached, 102 women enrolled in the study, 89 participants completed baseline measures, 79 completed 3-week follow-up measures, and 71 completed 3-month follow-up measures. Reasons provided for nonparticipation or attrition included time constraints and disinterest in participating in research studies.

### Procedure

Eligible breast cancer patients were identified by health-care providers and enrolled at treatment sessions. Informed consent was obtained at the time of enrolment. Participants completed baseline measures during the middle to later half of their treatment. Women receiving chemotherapy completed baseline measures at cycle 3 of 4 or 6 of 8. Women receiving 6–7 weeks of radiation therapy only completed baseline measures at week 4 or 5 of treatment. This schedule varied on occasion due to changes in treatment plans or scheduling difficulties. As such, time of initial assessment was adjusted for in primary study analyses. Participants were asked to complete follow-up measures via mail 3 weeks and 3 months following the last treatment session.

### Measures

#### Demographics and medical information

Basic demographic data and psychiatric history were obtained via participant self-report. Information regarding cancer stage, treatment, and current medications was abstracted from medical records.

#### Depressive symptoms

The Center for Epidemiological Studies Depression Scale (CES-D) is a 20-item scale used to assess depressive symptomatology (Radloff, 1977). Participants rated how often they experienced symptoms over the past week. A cutoff score of 16 has been established as indicative of probable clinical depression (Radloff, 1977; Ensel, 1986).

#### General anxiety

The seven-item Primary Care Evaluation of Mental Disorders Patient Health Questionnaire (PRIME-MD PHQ) anxiety scale assesses symptoms consistent with DSM-IV diagnostic criteria for generalised anxiety disorder (Spitzer *et al*, 1999). Participants rated how often they experienced each symptom over the past week.

#### Cancer-related anxiety

The Impact of Events Scale (IES) is a 15-item scale that assesses intrusive thoughts or rumination and attempts to avoid such thoughts (Horowitz *et al*, 1979). Participants were asked to rate frequency of intrusive thoughts about cancer and avoidance of these thoughts over the past week. A cutoff score of 19 has been established as indicative of a clinically significant stress response, while scores of 9–19 indicate a moderate stress response (Horowitz, 1982). The Concerns About Recurrence Scale (CARS) was used to assess worry about cancer recurrence (Vickberg, 2003). Women were asked to rate the frequency, potential for upset, consistency, and intensity of their worry about cancer recurrence.

#### Sources of distress

Participants were asked to rate to what extent a variety of factors had been a source of stress for them over the past week. Twelve items were chosen based on interviews with breast cancer survivors and the literature regarding sources of post-treatment stress among cancer patients (see Table 3).

#### Cancer-related symptoms

The Memorial Symptom Assessment Scale (MSAS) is a 32-item questionnaire that measures symptoms associated with cancer and its treatment (Portenoy *et al*, 1994). For each symptom experienced in the past week, participants rated its frequency, severity, and the extent to which it distressed them. Each symptom endorsed received a score calculated by averaging frequency, severity, and distress ratings, and the total score was the average of the symptom scores for all 32 symptoms.

#### Health-related quality of life

The Medical Outcomes Study Short Form 36 Version 2.0 (SF-36v2) is a 36-item scale, which measures health-related quality of life (Ware *et al*, 1993, 2000). Four of the eight SF-36v2 scales were used including physical functioning, role–physical (role limitations due to physical problems), bodily pain, and vitality scales.

### Statistical analyses

All variables were examined for outliers. Descriptive statistics and comparisons with normative samples were used to characterise outcome variables. Participants with missing data were included in analyses using time points for which they provided data. Mixed models analyses were conducted to examine changes in distress and quality of life measures across the three time points. In these models, the distress or quality of life outcome was entered as a repeated measure, and time at which baseline questionnaires were completed (measured in days prior to treatment completion) was entered as a covariate. Akaike's Information Criteria was used to select covariance matrices for each outcome based on fit and parsimony. Compound symmetry covariance matrices were used to model changes in distress measures (CES-D, IES, PRIME-MD, and CARS); autoregressive covariance structures were used to model changes in SF-36 subscales; and a heterogeneous autoregressive structure was used for the MSAS. When the overall test for change over time was significant, *post hoc* comparisons were performed to compare changes in distress or quality of life between time points.

Additional mixed model analyses examined the effects of demographic (age, relationship status, education, and income), psychiatric history (history of depression, history of anxiety, and current antidepressant use), and disease and treatment variables (disease stage, type of surgery, type of adjuvant treatment, length of treatment, and antioestrogen use) on depression, intrusion, and cancer-related worry, as well as on the trajectory of these measures over time. Separate models were tested for each predictor of interest with the distress measure as the repeated measure, the predictor and the interaction between time and the predictor as fixed effects, and time at which baseline questionnaires were completed as a covariate. Significant predictors were entered into a final multivariate model for each distress measure.

## RESULTS

### Participants

Demographic data and disease characteristics are presented in Table 1. Participants ranged in age from 32 to 89 years of age with a mean age of 55.0 years. All participants received adjuvant chemotherapy or radiation therapy, with 58% receiving both types of treatment. According to participants' self-report, 27% had been diagnosed with depression and 19% had been diagnosed with an anxiety disorder in the past. At the time of study entry, 28% were prescribed antidepressant medication.

Chi-square analyses and ANOVA were performed to examine whether women who dropped out of the study differed on demographic, disease, or outcome variables. Women who were enrolled in, but did not complete the entire study, did not differ from those who completed all time points on disease or demographic variables (all *P*-values exceeded 0.10) with one exception. Women who did not complete the study were more likely to be divorced or separated and less likely to be married or single, *χ*^2^=10.47, *P*=0.02. Women who completed at least one questionnaire packet but later dropped out did not differ from women who completed all time points on any study outcome variables at baseline (all *P*-values exceeded 0.10).

### Post-treatment distress

Means and standard deviations of distress measures are provided in Table 2. On average, participants in the present study were not highly distressed at any time point. CES-D mean scores following treatment were well under a cutoff score of 16, indicative of clinically significant depression and were consistent with scores reported for the general population (Radloff, 1977; Ensel, 1986; Hann *et al*, 1999). However, several participants exceeded the clinically significant cutoff score at baseline (19.3%), 3 weeks post-treatment (22.1%), and 3 months post-treatment (17.4%). Scores on the PRIME-MD also indicated relatively good adjustment on the whole, with women reporting on average that they experienced anxiety symptoms rarely or occasionally.

Cancer-specific distress was more predominant in the sample. Mean post-treatment scores on the IES were well under a cutoff score of 19, indicative of a clinically significant stress response (Horowitz, 1982), and few participants exceeded this cutoff on either intrusion or avoidance subscales at baseline (14.0 and 10.6%), 3 weeks post-treatment (9.0 and 10.5%), and 3 months post-treatment (8.6 and 11.6%). However, a greater number of women scored in the range of 9–19, indicative of a moderate stress response at baseline, (41.8 and 42.3%) 3 weeks post-treatment (38.4 and 38.3%), and 3 months post-treatment (34.3 and 36.2%). Worry about cancer recurrence, as assessed by the CARS, was also prevalent, with women reporting moderate levels of recurrence worry.

### Quality of life

Means and standard deviations of health-related quality of life measures are provided in Table 2. Women in the current study reported health-related quality of life that was generally on par with population norms of US women (SF-36.org, 1998). At baseline, participants' mean scores on physical functioning and role–physical (impairment in daily activities due to physical health) subscales were slightly lower than population norms (higher scores indicate better functioning), vitality scores were similar to norms, and bodily pain scores exceeded norms (indicating that the current sample reported less pain and pain-related impairment than the population at large). At follow-up time points, participants' mean scores on physical functioning and role–physical subscales increased to levels commensurate with population norms, while mean vitality scores increased to the extent that they exceeded norms.

### Changes in distress and quality of life over time

Mixed models indicated that there were no significant changes in depression, general anxiety, avoidance, or recurrence worry over the three study time points. Intrusion was the only distress variable that changed significantly, F(2,144)=3.48, *P*=0.034, declining significantly from baseline to 3 months post-treatment.

Memorial Symptom Assessment Scale symptom scores changed significantly over time, F(2,124)=8.98, *P*<0.001, declining from baseline to 3 weeks post-treatment, and then remaining steady. With respect to SF-36 quality of life domains, there were significant changes in physical functioning, F(2,148)=4.57, *P*=0.012; role–physical, F(2,149)=12.02, *P*<0.001; and vitality, F(2,155)=3.48, *P*=0.033. In all cases, quality of life improved over time, with contrasts revealing better quality of life at follow-up time points as compared to baseline. Table 2 provides follow-up contrasts between time points.

### Sources of post-treatment distress

Mean ratings of concerns are presented in Table 3. Following treatment, side effects or physical problems and fear of recurrence were the greatest sources of stress, with both rated to be moderately stressful on average. Getting back to normal or trying to create a ‘new normal’ were also notable sources of stress. Feeling like one has lost a safety net and not seeing health-care providers regularly were rated as ‘not at all’ to ‘a little bit’ stressful on average. Lack of support from family and friends was also not a significant concern.

### Univariate models predicting post-treatment distress

Demographic, psychiatric history, and disease and treatment variables were examined as potential predictors of distress and of the trajectory of distress over the course of the study using mixed models analyses.

**Figure 1:**
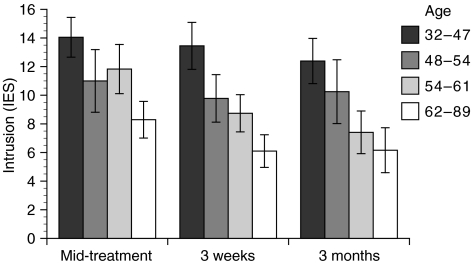
Age predicts intrusion, F(1,76)=12.12, *P*=0.001. Age is illustrated by quartiles.

**Figure 2:**
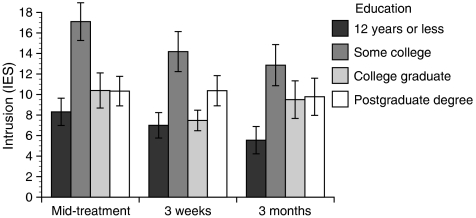
Education predicts intrusion, F(3,77)=6.77, *P*<0.001.

There was a main effect of age on all three distress outcomes, including depression, F(1,72)=9.62, *P*=0.003, intrusion, F(1,76)=12.12, *P*=0.001, and recurrence worry, F(1,82)=19.67, *P*<0.001. Younger age predicted greater distress on all measures (see Figure 1). Follow-up analyses investigated whether menopausal status might better account for this pattern; however, when both age and menopausal status were included in the models, only age significantly predicted distress. Education also significantly predicted all three distress outcomes: depression, F(3,74)=5.92, *P*=0.001, intrusion, F(3,77)=6.77, *P*<0.001, and recurrence worry, F(3,82)=6.50, *P*=0.001. Follow-up contrasts for education revealed that women who had completed some post-secondary education reported greater distress than did women who had completed 12 years of education or less and women who had completed a college or graduate degree (see Figure 2). However, age and education did not predict differential trajectories of distress over time.

**Figure 3:**
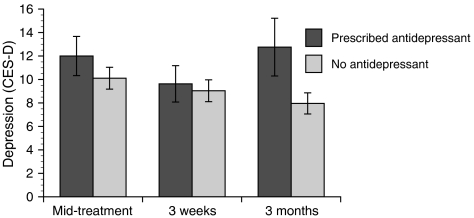
Psychiatric history predicts depression, F(1,80)=4.12, *P*=0.046, and the trajectory of depression over time, F(2,143)=3.50, *P*=0.033.

All psychiatric history variables examined were significant predictors of depression, including a history of depression, F(1,77)=13.37, *P*<0.001, a history of anxiety, F(1,79)=5.13, *P*=0.026, and being prescribed antidepressant medication, F(1,80)=4.12, *P*=0.046. Follow-up contrasts indicated that women with a history of depression or anxiety or those prescribed antidepressants were more likely to report depression over the course of the study. However, psychiatric history variables did not predict intrusion or recurrence worry. A history of anxiety and antidepressant use also predicted changes in depression over time, F(2,143)=3.52, *P*=0.032 and F(2,143)=3.50, *P*=0.033, respectively. Specifically, women with a history of anxiety showed a different trajectory of depression: their depressive symptoms decreased slightly from mid-treatment to 3 weeks post-treatment, and then increased steeply from 3 weeks to 3 months post-treatment. In contrast, women with no history of anxiety showed slightly declining depression over time such that differences between the two groups were most pronounced 3 months post-treatment. The same pattern of differential changes in depression was found for women who were prescribed antidepressants (see Figure 3).

**Figure 4:**
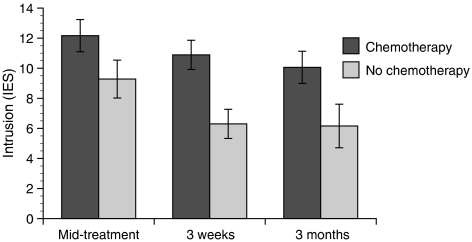
Adjuvant treatment predicts intrusion, F(1,81)=6.71, *P*=0.011.

In contrast to the psychiatric history variables, treatment-related variables significantly predicted intrusion and recurrence worry, F(1,81)=6.71, *P*=0.011 and F(1,84)=5.56, *P*=0.021, respectively, but not depressive symptomatology. Follow-up contrasts indicated that women who received chemotherapy reported greater anxiety than women who received radiation therapy only (see Figure 4). In addition, women who received a mastectomy reported greater recurrence worry than women who received a lumpectomy only, F(1,83)=6.45, *P*=0.012. Neither surgery nor adjuvant treatment predicted changes in intrusion or recurrence worry over time, however. Cancer stage and being prescribed an antioestrogen did not significantly predict anxiety.

### Multivariate models predicting distress

All individual variables found to be significant predictors of distress were entered into multivariate mixed models to determine which variables accounted for significant unique variance in predicting each outcome. Results are displayed in Table 4. In the model predicting depressive symptomatology, age, education, and a history of depression were significant predictors. The main effects of being prescribed antidepressant medication and a history of anxiety were not significant, nor were interactions between these variables and time. All individual predictors of intrusion remained significant when entered together into the model, including age, education, and adjuvant treatment. However, only age and education were significant predictors of recurrence worry.

## DISCUSSION

Breast cancer patients in the present study were remarkably well adjusted during the months following treatment as assessed by standard measures of psychiatric symptomatology and health-related quality of life. Consistent with study hypotheses, health-related quality of life improved significantly from mid-treatment to post-treatment, including improvements in physical functioning, role impairment, and vitality. Contrary to hypotheses, the post-treatment period was not, by most measures, a period of disrupted psychological adjustment. General distress measures were uniformly low across all study time points, and there was no significant increase in any indices of distress following the end of treatment. Instead, mean distress scores declined, and the decline was significant for intrusive thoughts.

These results are perplexing given the multitude of personal anecdotes (Mullan, 1985; McKinley, 2000), clinical observations (Rowland and Holland, 1990; Schnipper, 2001), and information collected via structured interview (Ward *et al*, 1992; Beisecker *et al*, 1997), suggesting that the post-treatment period is marked by distress, anxiety, transition, and disruption. However, results are consistent with a previous empirical investigation of breast cancer survivors (Deshields *et al*, 2005). It may be that clinical or anecdotal evidence leads to a bias towards overestimating the prevalence of post-treatment distress because generalisations are made based on a few remarkable cases. Another explanation for the discrepancy between our findings and previous nonempirical work is the possibility that measures of depression and general anxiety do not capture the type of distress patients are reporting and clinicians are noting. Consistent with this idea, participants were more likely to report cancer-specific distress and worry. In particular, worry about a cancer recurrence was common; women reported moderate levels of recurrence worry and fear of a recurrence was one of the top-rated sources of distress at both post-treatment time points.

The finding that physical problems related to cancer and treatment was also a top source of distress following treatment was unexpected in light of the good quality of life women reported following treatment. It may be that distress related to physical problems is not indicative of high levels of physical disability, but may reflect survivors' expectations regarding recovery. Previous work suggests that women often do not anticipate ongoing treatment-related problems but instead expect to return to ‘normal’ shortly after treatment ends (Beisecker *et al*, 1997; Stanton *et al*, 2005). This is consistent with the current study's finding that ‘trying to get back to normal life’ was another significant source of distress for women in the present study, as was ‘creating a ‘new normal’’. Returning to ‘normal’ may not be easy or realistic, and women may need additional education regarding longer term physical and emotional effects.

It was hypothesised that worry about a cancer recurrence, in combination with the loss of the ‘safety net’ of regularly attending cancer treatments, may be one of the most significant sources of distress for patients. Although fear of recurrence was prevalent in the current sample, the loss of a safety net was not a significant source of distress for most women. Moreover, contrary to expectations, loss of instrumental and emotional support from family and friends or from health-care providers was rated lowest on a list of potential stressors.

While the majority of participants exhibited good adjustment following treatment, some women were at risk for elevated distress. In particular, younger women were significantly more likely to experience depression, anxiety, and cancer-related distress, a pattern that is consistent with previous studies of breast cancer survivors (Wenzel *et al*, 1999; Arndt *et al*, 2004). Age was the most robust predictor of distress and remained significant in models with multiple predictors. Developmental theorists have proposed that ‘off-time’ life events occurring outside of typical age ranges are more likely to be distressing or even traumatic (Neugarten and Hagestad, 1976). Younger breast cancer patients may be less likely to have considered the possibility of developing a serious illness or to have peers with health problems. Greater demands in the areas of work or parenting and fewer coping resources may also make cancer treatment particularly stressful for younger women. Women who had completed some college education were also more distressed than women with both more and less education, and this remained true in models adjusting for age. It may be that women with higher levels of education are better able to utilise medical information or communicate with providers, but it is unclear why women with the least amount of education would have lower distress.

Women with a self-reported history of anxiety or depression and those who were taking antidepressant medication were most likely to experience elevated depression. Moreover, depression in women with a psychiatric history increased notably between 3 weeks and 3 months post-treatment, such that differences between these women and other participants were most striking 3 months post-treatment. However, this risk did not extend to cancer-related anxiety. It appears that a history of emotional disturbance uniquely places women at greater risk for general depressive symptomatology, and that this risk increases during the months following treatment.

In contrast, women who received more extensive treatment were at greater risk for cancer-related anxiety following treatment but did not report elevated depressive symptomatology. Specifically, women who received chemotherapy reported greater cancer-related intrusion and recurrence worry as compared to women who received radiation therapy only. It may be that the longer duration and more severe side effect profile associated with chemotherapy as compared to radiation therapy results in greater cancer-related anxiety. Women undergoing chemotherapy are also more likely to have severe disease and a greater risk of recurrence, but cancer stage was not related to cancer-related anxiety. Women who underwent a partial or total mastectomy also endorsed greater recurrence worry than did women who received a lumpectomy. It may be that women who are more anxious about a recurrence opt for a mastectomy, but results suggest that having a mastectomy does not alleviate or normalise recurrence worry.

Limitations of the present study include the homogenous nature of the sample; almost all participants were Caucasian, and the vast majority were married and well educated. Therefore, generalisations to other populations of breast cancer survivors may be limited. In addition, it is unclear whether timing of the baseline assessment was ideal. Because it was timed relative to treatment plans, baseline measures were completed at varying lengths of time since diagnosis and prior to the end of treatment. Ideally, one would compare post-treatment adjustment to adjustment prior to diagnosis. Given the near impossibility of conducting such a study, it may be beneficial to measure distress at additional points such as just after diagnosis but prior to treatment and on the last day of treatment to provide a fuller context for interpreting of the nature and extent of distress following treatment.

In summary, most breast cancer survivors do not experience a period of disrupted adjustment with respect to general psychiatric symptoms and traditional quality of life measures. This does not mean, however, that cancer survivors' lives return to normal after treatment ends. To the contrary, many women experience at least moderate cancer-related anxiety and report significant concerns about ongoing physical symptoms, the possibility of a cancer recurrence, and how to go about rebuilding a ‘new normal’. Difficulty obtaining one's previous or new ‘normal’, both physically and otherwise, in combination with the realisation that cancer may return to disrupt one's life again, best characterises the type of distress reported by women in the present study. These specific indicators of distress may be more relevant than general distress indices following treatment and should therefore be the subject of attention by health-care providers and researchers.

Our results also suggest that particular subsets of breast cancer survivors may be at risk for elevated distress following treatment, most notably younger women. Women with a history of emotional disturbance are at particular risk for a nonspecific depressive response developing later in the post-treatment period, while those who receive more extensive treatment are at risk for more acute, cancer-specific anxiety. Health-care providers should be aware of these risk factors, and tailored interventions targeting the vulnerabilities associated with each may help breast cancer survivors to navigate the post-treatment period and create a satisfying ‘new normal’.

## Figures and Tables

**Table 1:** Sample characteristics

	**Percentage of sample**
*Relationship status*	
Married or living with partner	73.8
Single	5.7
Divorced or separated	12.5
Widowed	7.9
	
*Education*	
High school graduate or less	31.8
Some college	22.7
College graduate	21.6
Postgraduate degree	23.9
	
*Income*	
<$25 000	20.6
$25 001–40 000	12.3
$40 001–55 000	13.7
$55 001–70 000	19.2
>$70 000	34.2
	
*Ethnicity*	
Caucasian	93.2
African American	2.3
Asian	1.1
Native American	1.1
Other	2.3
	
*Cancer stage*	
0	5.7
I	33.3
II	47.1
III	13.8
	
*Treatment* a	
Mastectomy	27.3
Lumpectomy	79.5
Chemotherapy	71.6
Radiation therapy	86.4
Hormonal therapy	77.0

a Treatment categories are not mutually exclusive.

**Table 2:** Mean distress and quality of life scores over time

	**Baseline**	**3 weeks**	**3 months**	**Contrasts**
*Distress measures*				
CES-D	10.63	9.18	9.14	
	(7.62)	(6.92)	(7.76)	
IES				
Intrusion^a^	11.32	9.52	9.00	T1>T3
	(7.81)	(6.78)	(7.45)	
Avoidance	11.08	9.99	10.06	
	(7.08)	(6.69)	(7.44)	
PRIME-MD				
Anxiety	11.18	11.38	10.84	
	(2.86)	(3.09)	(3.35)	
				
CARS	11.78	12.40	11.50	
	(5.06)	(5.05)	(4.64)	
				
*Quality of life measures*				
MSAS				
Total symptoms^a^	0.69	0.51	0.50	T1>T2
	(0.47)	(0.34)	(0.31)	T1>T3
SF-36				
Physical function^a^	74.37	78.59	81.88	T1<T2
	(23.02)	(20.78)	(21.27)	T1<T3
Role–physical^a^	60.85	71.63	77.45	T1<T2
	(25.88)	(24.19)	(23.95)	T1<T3
Bodily pain	82.05	80.77	81.01	
	(19.54)	(18.57)	(18.64)	
Vitality^a^	53.05	58.09	61.68	T1<T3
	(19.90)	(20.60)	(21.14)	

CARS=Concerns About Recurrence Scale; CES-D=Center for Epidemiological Studies Depression Scale; IES=Impact of Events Scale; MSAS=Memorial Symptom Assessment Scale; PRIME-MD=Primary Care Evaluation of Mental Disorders; SF-36=Medical Outcomes Study Short Form 36; T1=baseline; T2=3 weeks post-treatment; T3=3 months post-treatment.

Note: Standard deviations are enclosed in parentheses. Contrasts were performed only if the overall F-test was significant at *P*<0.05.

a Overall F-test was significant at *P*<0.05.

**Table 3:** Mean ratings of post-treatment sources of stress

**Sources of stress**	**3 weeks**	**3 months**
Fear of a cancer recurrence	2.58 (1.14)	2.48 (1.16)
Side effects or physical problems related to cancer and treatment	2.35 (1.19)	2.54 (1.27)
Trying to get back to normal life now that treatment has ended	2.46 (1.34)	2.25 (1.42)
Creating a ‘new normal’ now that treatment has ended	2.22 (1.25)	2.22 (1.30)
Worry about the impact of cancer on my family	2.15 (1.22)	2.18 (1.97
Feeling unsure what to do for my health or to prevent a cancer recurrence	2.16 (1.24)	1.96 (1.09)
My emotions or emotional well-being	1.91 (0.96)	1.84 (0.98)
Concerns about ability to fulfill responsibilities at work or home	1.79 (1.08)	1.72 (1.03)
Feeling like I have lost a ‘safety net’ now that treatment has ended	1.59 (0.91)	1.54 (0.88)
Not seeing oncologist and health-care staff regularly now that treatment has ended	1.34 (0.81)	1.39 (0.75)
Not getting the assistance I would like from family or friends	1.38 (0.80)	1.33 (0.63)
Not getting the emotional support I would like from family or friends	1.31 (0.71)	1.25 (0.53)

Note: Standard deviations are enclosed in parentheses. Ratings ranged from 1 ‘not at all’ to 5 ‘very much’.

**Table 4:** Multivariate distress models

	***F***	***P***
*CES-D depression model*		
Age	8.68	0.004
Education	4.66	0.005
History of depression	4.90	0.03
History of anxiety	0.42	0.52
Prescribed antidepressant	0.14	0.70
History of anxiety × time	1.29	0.28
Prescribed antidepressant × time	1.49	0.23
		
*IES intrusion model*		
Age	6.24	0.02
Education	8.43	<0.001
Chemotherapy	5.95	0.02
		
*CARS recurrence worry model*		
Age	9.90	0.002
Education	7.65	<0.001
Chemotherapy	2.66	0.11
Mastectomy	0.79	0.38

CARS=Concerns About Recurrence Scale; CES-D=Center for Epidemiological Studies Depression Scale; IES=Impact of Events Scale.

Note: All models adjusted for time of baseline assessment.
